# Factors analysis of lower probability of receiving bystander CPR in females: a web-based survey

**DOI:** 10.1186/s12872-025-04709-5

**Published:** 2025-04-08

**Authors:** Wangxinjun Cheng, Jingshuang Liu, Chufan Zhou, Xuzhen Wang

**Affiliations:** 1 Department of Intensive Care Unit, the First Affiliated Hospital of Nanchang University, Nanchang, Jiangxi China; 2 Queen Mary College, Nanchang University, Nanchang, Jiangxi China; 3 West China School of Public Health and West China Forth Hospital, Sichuan University, Chengdu, Sichuan China

**Keywords:** Female, Web-based survey, CPR, Bystander

## Abstract

**Background:**

Women are less likely to receive bystander cardiopulmonary resuscitation (CPR) during out-of-hospital cardiac arrest (OHCA) compared to men. This study aims to identify the factors influencing the willingness to perform CPR on women, providing insights to improve training and public awareness.

**Methods:**

A cross-sectional web-based survey was conducted among medical and non-medical populations in southeastern China. The questionnaire assessed demographics, CPR training experience, and attitudes toward gender-related CPR concerns. A total of 450 responses were collected, with 433 valid responses included after quality control. Statistical analyses were performed using R4.3.2 to evaluate the impact of gender, age, occupation, and education on CPR willingness.

**Results:**

Women exhibited a higher willingness to perform CPR on female victims compared to men. Many male respondents hesitated due to concerns about physical contact, particularly regarding removing clothing during resuscitation. Younger individuals (18–35 and 36–50 years) showed greater willingness to provide CPR than older respondents (51–75 years), who were more cautious due to privacy concerns and traditional beliefs. Healthcare professionals and non-medical workers were more likely to perform CPR than medical students, who, despite receiving CPR training, expressed hesitation due to a lack of confidence and practical experience. Higher education levels were associated with increased willingness to perform CPR on women, with postgraduate respondents being the most willing. Additionally, most participants had never practiced on female CPR mannequins, despite widespread support—especially among women—for incorporating female models into training.

**Conclusion:**

The lower likelihood of women receiving CPR is influenced by gender bias, societal norms, and training limitations. Addressing this issue requires public education to eliminate gender-based hesitation, improvements in CPR training programs to include female mannequins, and enhanced legal protections to reduce rescuer concerns. These measures can be combined with other key factors such as community-wide CPR training programs and increasing the availability of automated external defibrillators (AEDs) to help promote equity in access to life-saving interventions. Targeted interventions can promote gender equity in emergency response, ultimately improving survival outcomes for women.

## Introduction

Sudden cardiac arrest (SCA) continues to be a significant global health issue, particularly when it occurs outside of hospital settings. It is the primary cause of death in Western countries and the leading cause of death related to cardiovascular disease. In the United States and Western Europe, SCA accounts for 15–20% of all natural deaths in adults and 50% of all cardiovascular-related deaths. Furthermore, SCA is responsible for approximately half of all cases of coronary heart disease [1, 2]. In China, 544, 000 sudden cardiac deaths occur each year, with a survival rate of less than 1% [3]. In addition, it is worth noting that in cases of cardiac arrest, the quality of cardiopulmonary resuscitation (CPR) plays a crucial role in determining the chances of patient survival [4]. CPR, or cardiopulmonary resuscitation, is an emergency procedure performed on individuals who are unresponsive and not breathing due to cardiac arrest. It involves a combination of chest compressions and rescue breathing, usually in a ratio of 30 compressions to 2 breaths [5]. CPR is crucial in improving blood circulation and maintaining brain function until specialized equipment, such as an automated external defibrillator (AED), can be utilized [6, 7]. Numerous studies have demonstrated that bystander-initiated CPR significantly enhances a patient’s chance of survival, thereby playing a crucial role in improving the prognosis of out-of-hospital cardiac arrest (OHCA) [8, 9].

Unfortunately, several studies have shown that women have a lower likelihood of receiving effective CPR during OHCA compared to men, resulting in a lower probability of successful resuscitation and significantly lower survival rates to hospital discharge [10, 11]. The sex difference in survival rates may be attributed to a variety of underlying factors, including social factors and resistance to touching women’s secondary sexual characteristics such as the chest, which may be considered embarrassing or offensive. Additionally, the use of male manikins in practice and training may result in a subconscious preference for performing CPR on male individuals. There may also be a lack of knowledge on how to perform CPR on women, including the proper methods for removing intimate clothing. These factors may contribute to disparities in the receipt and quality of CPR for women in cases of OHCA [12–14]. At the same time, the CPR training rate of students in China is relatively low compared with other countries [15–18]. Overall, the survival rate of cardiac arrest in China is also much lower than that in many countries [15]. These factors significantly reduce the likelihood of women receiving CPR in China. This is a critical issue that directly impacts the lives and health of women, making it of utmost importance.

As society progresses and people’s attitudes evolve, there is a need to explore the factors influencing bystanders’ gender considerations when performing CPR on patients experiencing cardiac arrest. Surprisingly, there is a lack of research investigating these influencing factors through questionnaires. Therefore, this study aims to examine the public attitudes in Chinese society, with the goal of understanding the specific reasons behind the impact of patients’ gender on bystanders’ willingness to initiate CPR. The findings of this study may provide valuable insights and actionable directions to enhance the survival rate of female CPR recipients.

## Method

This study employed a cross-sectional observational design using a web-based survey. The survey was released through Questionnaire Star, which features a quick response code (QR code) and was administered via a web-based platform. Medical staff, medical students, and the general public in Jiangxi province were primarily invited to complete the survey.The questionnaire was developed collaboratively by the authors and pilot tested in advance and revised based on feedback from the pre-experiment. It included 18 closed-ended questions organized into three sections. The first section focused on demographics (question 1–5) and collected basic information such as gender, age, and occupation. The second section concentrated on the basic practice of CPR (question 6–13), inquiring about participants’ CPR training, their ability to perform CPR, and the willingness of both men and women to engage in CPR. The final section comprised a survey on perceptions regarding the success rate of CPR in women, addressing issues such as public rescue scenarios, removing clothing, and hesitations related to consciousness. Respondents’ attitudes and behaviors toward performing CPR on women were assessed with a 7-item Likert scale, measured on a five-point scale from low to high. Other questions were on a five-point scale ranging from strongly agree to strongly disagree. In this study, 1 means strongly disagree, 2 means disagree, 3 means neutral, 4 means agree, and 5 means strongly agree.

The sample size of the questionnaire is determined by the formula $\:n=({\frac{{Z}_{\frac{\alpha\:}{2}}}{\delta\:})}^{2}\bullet\:\pi\:(1-\pi\:)$, where n is the sample size, Z_(α/2) is the confidence level, δ is the permissible error, and π is the population rate. Assuming a very large population size, setting a confidence level of 95% and a confidence interval of 5%, the calculated minimum required sample size is at least 267. Considering a 10% rate of invalid questionnaires, the final minimum sample size to be included is proposed to be 294.

The present study conducted differential analysis on various influencing factors (such as gender, occupation, age group, and educational level) on each questionnaire item in the general population and individuals familiar with CPR. The results showing differences were then tabulated for detailed analysis and discussion.

The survey was hosted by the Internet-based survey site Questionnaire Star, which supports anonymous, self-managed participation. Consent was obtained by submitting the complete survey. The survey was conducted from March to October 2023. The completed survey data were entered into R4.3.2 for statistical analysis.

## Results

### Demographic distribution

In this survey, a total of 450 questionnaires were meticulously collected, ensuring the integrity and quality of the data. Rigorous quality control measures were implemented, resulting in the exclusion of 17 questionnaires that exhibited signs of incomplete or rushed responses, such as those completed in less than 1 min. Subsequently, 433 questionnaires remained eligible for inclusion in the data analysis phase, representing a robust effective recovery rate of 96%.

The demographic composition of the respondents was diverse, with a balanced representation of genders. Specifically, 47.58% of the participants identified as male, while 52.42% identified as female, indicating a relatively equal distribution across genders. The age distribution reveals that the vast majority of respondents fall within the 18–35 age group, accounting for 68.36% of the total, with a total of 296 individuals. In this study, the majority of respondents are affiliated with the medical industry, particularly medical students, constituting 59.12% of the total, with 256 individuals, representing over half of the proportion. The distribution of education levels indicates that respondents generally have higher educational backgrounds, with individuals holding a Bachelor’s degree comprising 88.91% of the total, totaling 385 individuals. Detailed basic information is shown in Table 1.

**Table 1:** Demographic characteristics of the sample(*N* = 433)

Relevant factors	n	%	Cumulative %
**Gender**			
** Male**	206	47.58%	47.58%
** Female**	227	52.42%	100.00%
**Age**			
** 18–35 years**	296	68.36%	68.36%
** 36–50 years**	84	19.40%	87.76%
** 51–75 years**	53	12.24%	100.00%
**Occupation**			
** Medical students**	256	59.12%	59.12%
** Medical industry workers**	129	29.79%	88.91%
** Non-medical industry workers**	48	11.09%	100.00%
**Education Level**			
** Middle School**	13	3.00%	3.00%
** Bachelor's Degree**	385	88.91%	91.92%
** Postgraduate and above**	35	8.08%	100.00%

### Multifactorial analysis of the willingness to perform CPR on women

The willingness to perform CPR on women in emergency situations by the general public is influenced by various factors such as gender, age, occupation, and education level. Table 2 summarizes the frequencies and proportions of individuals of different genders, age groups, occupations, and education levels who are willing to perform CPR on women in emergency situations. Compared to males, females show a slightly higher overall proportion of willingness to perform CPR on women, including both willing and very willing individuals. The predominant choice among individuals of different age groups regarding willingness to perform CPR on females was option “willing”. However, upon comparing the three age groups, it was observed that the age groups of 18–35 years and 36–50 years were more inclined towards performing CPR on females compared to the age group of 51–75 years. The proportions of individuals willing to perform CPR on females in these three age groups were 70.6%, 75%, and 58.5%, respectively. The proportions of willingness to perform CPR on women are similar between healthcare professionals and non-healthcare professionals, with 75.77% and 77.08% of each respective total population. Surprisingly, medical students show the lowest proportion of willingness to perform CPR on women at approximately 68.75% among the three groups. Among healthcare professionals, the proportion of individuals who are very willing is the highest at 27.71%. There are differences in the willingness to perform CPR on women among groups with different education levels, with higher education levels correlating with higher willingness to perform CPR on women. The highest proportion of.

**Table 2:** Analysis of general factors influencing the willingness of different populations to perform CPR on females

Relevant factors	Degree of Willingness					Total
	Strongly unwilling (*n*, % of total)	Unwilling (*n*, % of total)	Neutral (*n*, % of total)	Willing (*n*, % of total)	Strongly willing (*n*, % of total)	
Gender						
Male	9(4.37%)	11(5.34%)	43(20.87%)	108(52.43%)	35(16.99%)	206(100%)
Female	10(4.85%)	6(2.91%)	51(24.76%)	116(56.31%)	44(21.36%)	227(100%)
Age						
18–35 years	1(0.34%)	6(2.03%)	80(27.03%)	165(55.74%)	44(14.86%)	296(100%)
36–50 years	7(8.33%)	7(8.33%)	7(8.33%)	42(50.00%)	21(25.00%)	84(100%)
51–75 years	11(20.75%)	4(7.55%)	7(13.21%)	17(32.08%)	14(26.42%)	53(100%)
Occupation						
Medical students	1(0.39%)	4(1.56%)	75(29.30%)	138(53.91%)	38(14.84%)	256(100%)
Medical industry workers	17(13.18%)	8(6.20%)	14(10.85%)	62(48.06%)	28(27.71%)	129(100%)
Non-medical industry workers	1(2.08%)	5(10.42%)	5(10.42%)	24(50%)	13(27.08%)	48(100%)
Education Level						
Middle School	1(7.69%)	1(7.69%)	3(23.08%)	5(38.46%)	3(23.08%)	13(100%)
Bachelor’s Degree	17(4.42%)	14(3.64%)	86(22.34%)	200(51.95%)	68(17.66%)	385(100%)
Postgraduate and above	1(2.86%)	2(5.71%)	5(14.29%)	19(54.29%)	8(22.86%)	35(100%)

individuals willing to perform CPR on women is among those with postgraduate education or above at 77.15%, followed by those with undergraduate education at 69.61%, and the lowest proportion among individuals with secondary education at 61.54%. The detailed frequencies and proportions for each option within each group can be observed in Table 2.

### The influence of gender factors on the choices of the general population

In the general population, there exists a significant difference in attitudes between males and females regarding the impact of female breasts on CPR procedures (*p* < 0.05). The majority of females (approximately 41.41%) do not believe (including strongly disagree and disagree options) that female breasts would affect CPR, while only 21.85% of males do not believe that female breasts would affect CPR. The proportion of females holding a disagreement stance in all options is the highest among this group (32.06%), while the majority of males opt for a neutral stance (38.83%).

In the general population, both men and women have the highest proportion of individuals who disagree with the notion that CPR is more difficult to perform on men than on women (women: 49.78%, men: 39.32%). However, there is a significant difference in the choices between the two groups (*P* < 0.05), with women having a 13.1% higher overall proportion of individuals opposing this view compared to men. The overall opposition includes the total number of individuals who strongly disagree and disagree with this notion.

In the general population, both men and women generally agree on the issue of “women being less likely to receive CPR rescue in public due to social and cultural factors.” The total number of men who agree reached 58.74% of the total male respondents, while the total number of women who agree reached 55.94% of the total female respondents. However, there is a significant difference in the choices of these two groups regarding this option (*P* < 0.05). The proportion of women choosing the disagree option (23.79%) is significantly higher than the proportion of men choosing this option (14.08%). Furthermore, men seem to exhibit a more intense level of agreement compared to women, as the proportion of men selecting the strongly agree option reached 22.82% of the male population, while the proportion of women selecting the strongly agree option was only 12.33% of the female population.

There is a significant difference in attitudes between males and females in the general population regarding the removal of clothing for performing CPR on women (*P* < 0.05). 42.73% of female participants did not consider this embarrassing or offensive, while only 29.61% of male participants did not find it embarrassing. The proportion of males who maintained a neutral attitude (24.76%) was significantly higher than the proportion of females who chose this option (16.74%). Furthermore, in response to the question of whether removing clothing to perform CPR on women is embarrassing and offensive, the proportion of males who selected “agree” and “strongly agree” among the five options was slightly higher than the proportion of females who selected these two options.

**Fig. 1:**
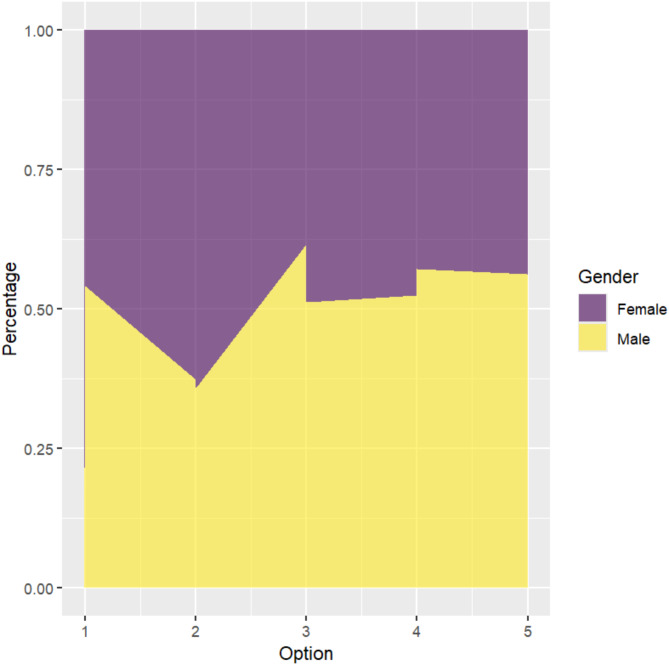
Analysis of the influence of gender on the choices of the general populationThe impact of age factors on the choices of the general population

In the general population, there is a difference in attitudes towards performing CPR on individuals of the opposite gender between males and females (*P* < 0.05). The majority of both males and females would hesitate in such a situation (males: 55.82%, females: 44.06%), but the proportion of males hesitating is slightly higher than that of females. We present the data visualization in Fig. 1.

There is a significant difference in attitudes towards whether the female breast affects the performance of CPR among different age groups (*P* < 0.05). The proportion of individuals aged 36 and above who strongly agree that the female breast affects the performance of CPR is significantly higher than the proportion of individuals aged 36 and below who choose this option. Additionally, the proportion of individuals in the 36–50 age group and 51–75 age group who agree with this viewpoint is also significantly higher than that of the 18–35 age group (36–50 age group: 57.14%, 51–75 age group: 54.71%, 18–35 age group: 27.7%).

In the general population, there is a significant difference in perceptions of the difficulty of performing CPR on males versus females among different age groups (*P* < 0.05). The most common choice among the 18–35 age group is to disagree that performing CPR on males is more difficult than on females, while individuals aged 36 and above most frequently choose to agree that performing CPR on males is more challenging. The proportion of individuals aged 36 and above who agree that performing CPR on males is more difficult than on females is significantly higher than that of individuals aged 36 and below (36–50 age group: 51.19%, 51–75 age group: 56.6%, 18–35 age group: 12.16%).

There are variations in attitudes towards immediately performing CPR in the event of sudden cardiac arrest in women across different age groups (*P* < 0.05). In the 18–35 age group, the attitudes are relatively balanced, with “agree,” “neutral,” and “disagree” options closely distributed at 34.80%, 23.99%, and 28.04% respectively. However, the overall agreement rate (45.27%) surpasses the overall disagreement rate (30.74%). Nevertheless, the proportion of strong agreement is the lowest across all three age groups (10.47%). In the 36–50 age group, “agree” holds the highest proportion at 39.29%, followed by “strongly agree” at 26.19%. Additionally, the proportions of “disagree” and “strongly disagree” in this age group are relatively low at 14.29% and 7.14% respectively, indicating a lesser degree of dissenting opinions. In the 51–75 age group, the combined proportion of “strongly agree” and “agree” reaches 49.06%, nearly half of the population. Overall, in all age groups, the total of “agree” and “strongly agree” exceeds that of “disagree” and “strongly disagree,” demonstrating that a majority of individuals support taking immediate life-saving measures in the event of sudden cardiac arrest in women.

There are differences in attitudes among different age groups regarding considering patient privacy, especially in covering the exposed chest of the patient during CPR (*P* < 0.05). Overall, the total of “agree” and “strongly agree” predominates across all age groups. In the 51–75 age group, the combined proportion of “agree” and “strongly agree” is 58.49%, which is the lowest among the three age groups (18–35: 79.05%, 36–50: 65.48%). The populations of “disagree” and “strongly disagree” are minorities in each age group, but the proportions of these two options are relatively higher in the 51–75 age group.

**Fig. 2:**
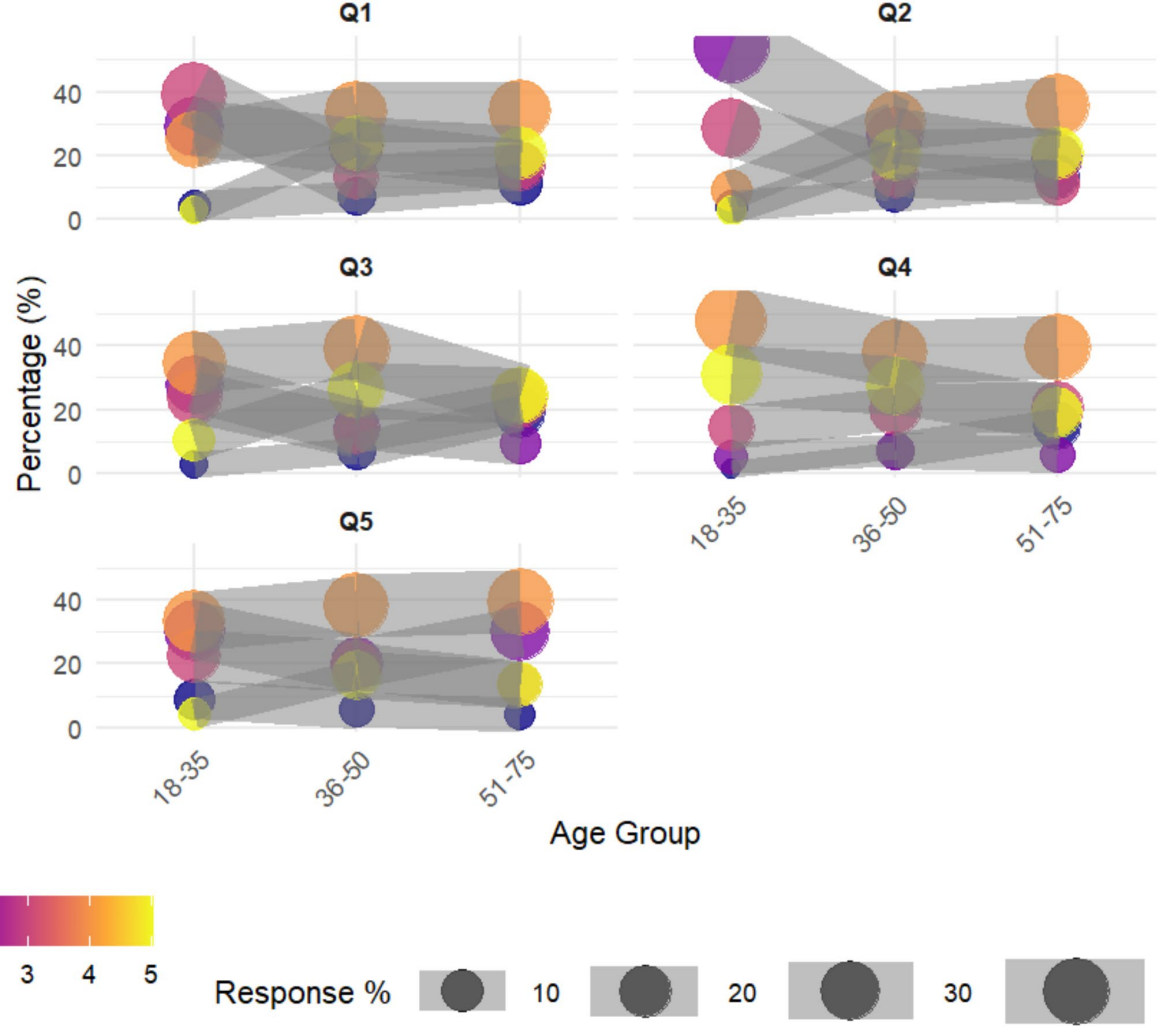
Analysis of the influence of age on the choices of the general populationThe impact of educational attainment on the choices of the general population

Differences were observed among various age groups in their attitudes towards removing clothing from women during CPR, with a significance level of *P* < 0.05. The proportion of individuals in each age group selecting the “agree” option was highest compared to other options, with percentages of 33.45% for the 18–35 age group, 38.10% for the 36–50 age group, and 39.62% for the 51–75 age group. However, significant disparities were noted, particularly between the 18–35 age group and those above 36 years old, in extreme opinions. The older age groups exhibited a more distinct stance, with 16.67% of the 36–50 age group strongly agreeing that removing a woman’s clothing during CPR is embarrassing and offensive, and 13.21% of the 51–75 age group sharing the same sentiment. In contrast, only 4.39% of the 18–35 age group strongly agreed with this viewpoint We present the data visualization in Fig. 2.

**Fig. 3:**
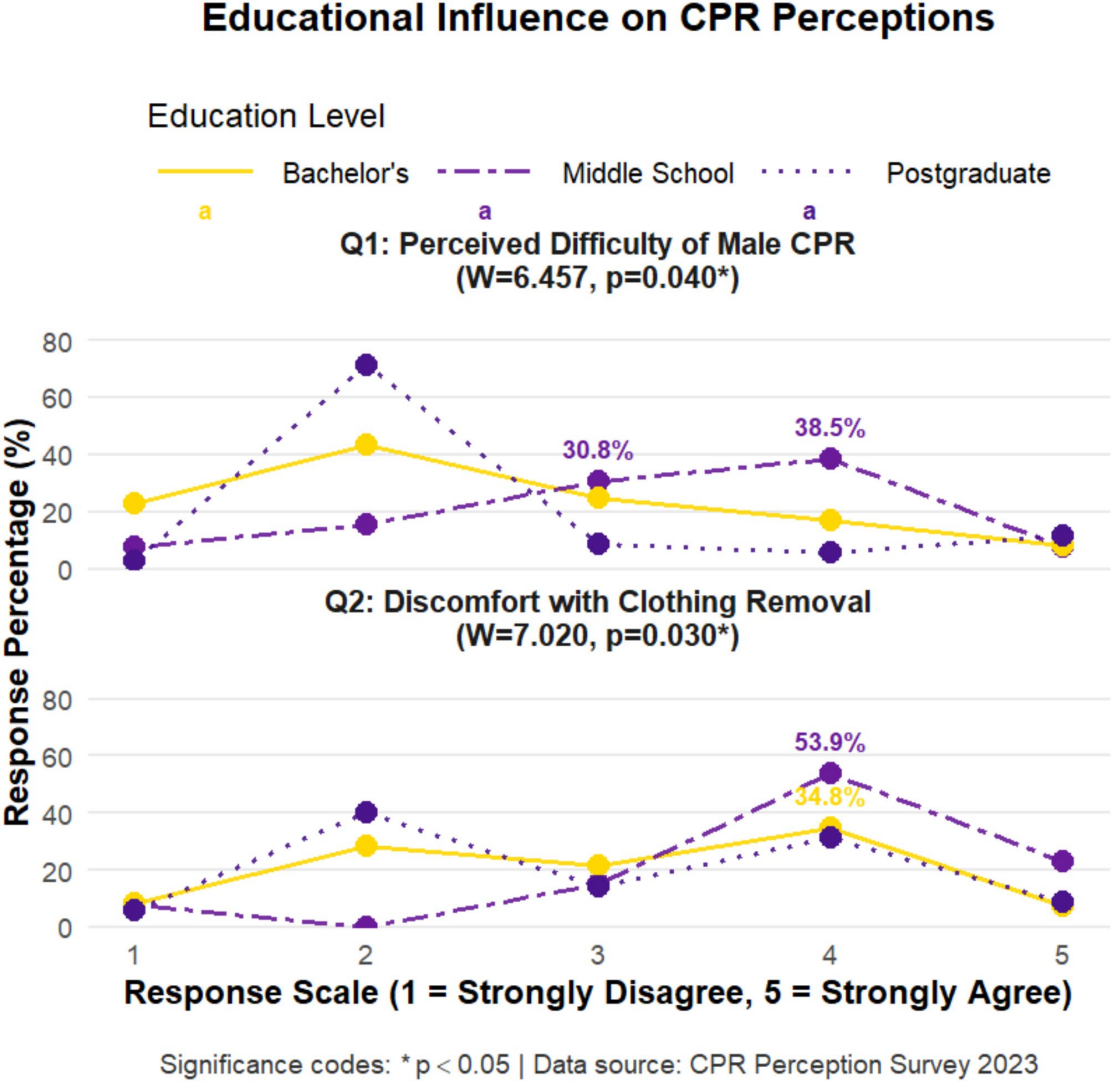
Analysis of the influence of education level on the choices of the general population

A disparity exists in perceived CPR difficulty between males and females among individuals with different educational levels. More individuals with postgraduate education (74.29%) disagree that female chest anatomy affects CPR performance than those with undergraduate (66.38%) or high school (23.07%) educations. A significant difference (*P* < 0.05) exists in attitudes toward removing women’s clothing during CPR based on education level. Most postgraduate-educated individuals do not find removing a woman’s clothing during CPR awkward or offensive, while undergraduate students are slightly more likely to agree (47.27%) than disagree (36.62%). Most high school students (76.93%) find removing a woman’s clothing during CPR awkward and offensive. Frequencies and proportions for each question, by education level, are showed in Fig. 3.

### The influence of occupation factors on the choices of the general population

**Fig. 4:**
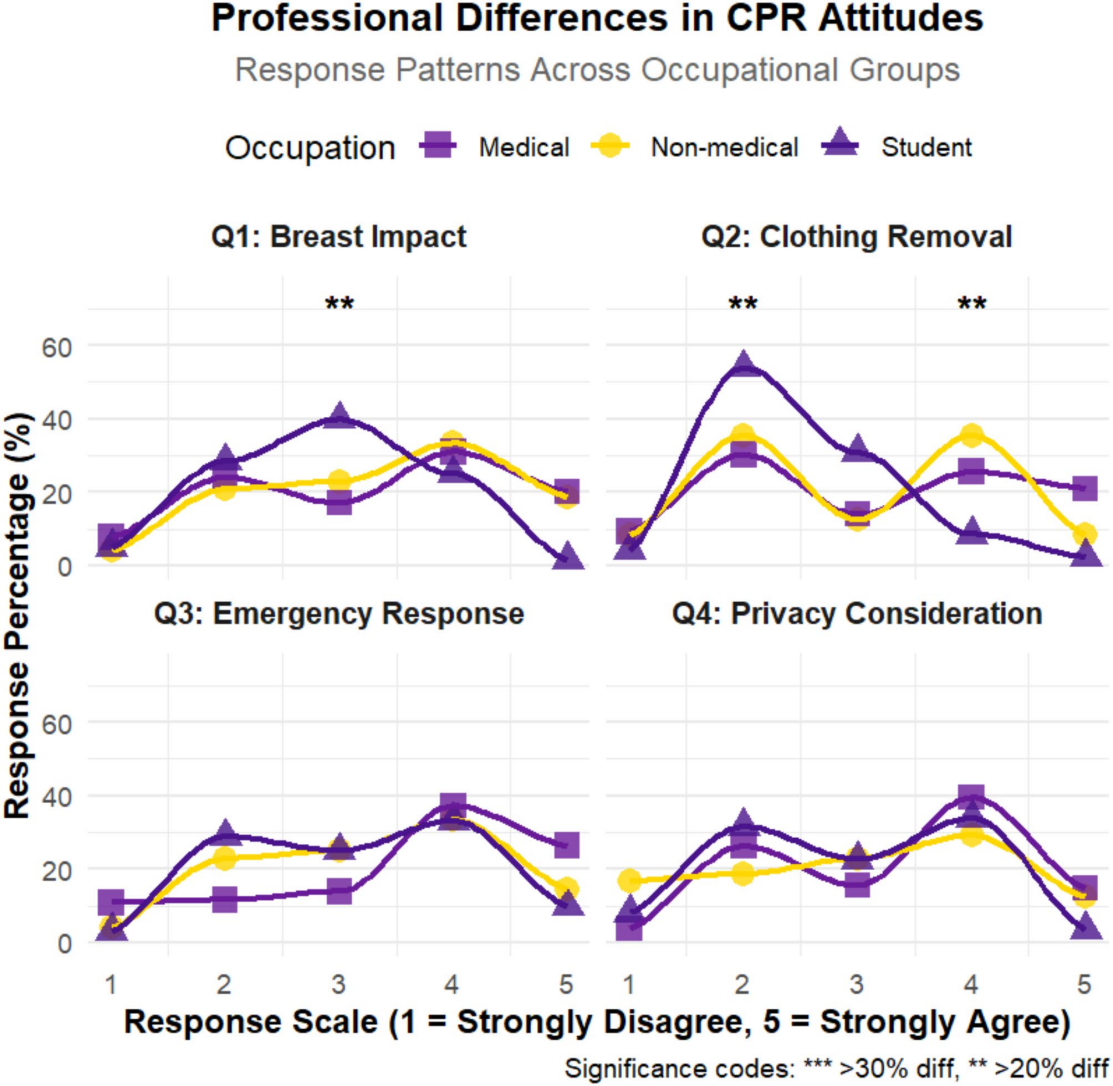
Analysis of the influence of occupation on the choices of the general population

Discrepancies exist in how various professional and social groups perceive the impact of female breasts on CPR procedures. Only 26.56% of medical students agree that female breasts could impact CPR procedures, compared to 51.17% and 52.08% of healthcare professionals and non-healthcare industry workers, respectively. Significant differences (*P* < 0.05) exist in perceptions of CPR difficulty between genders across various professional and social groups, with medical students less likely to agree that CPR is more difficult on males (10.93%) than on females, compared to non-healthcare industry workers (43.75%) and healthcare professionals (46.51%). Attitudes towards immediately performing CPR on women with sudden cardiac arrest vary by occupation and social group (*P* < 0.05), with medical students exhibiting a more balanced distribution of opinions. Regarding undressing women for CPR, medical students are split, with a slight majority disagreeing that it is embarrassing (37.5% agree vs. 39.84% disagree). Analysis of frequencies and proportions for each option by occupation are provided in Fig. 4. In view of the professional nature of the setting of some questions, we selected the data of people who had learned and could do CPR for a more detailed analysis, a total of 285.

### The impact of educational training on women’s acquisition of CPR skills

#### The impact of the mastery of CPR skills on the choice of questions

Comparison with above data shows that many factors influencing the CPR-trained population are no longer significant for certain issues (*P* > 0.05). For example, responses to questions about CPR difficulty on males versus females and societal factors affecting CPR assistance for females no longer differ by gender. Similarly, there is no significant difference in responses to questions about immediate CPR on women in sudden cardiac arrest across different age groups. However, certain factors remain significant among the CPR-trained population. Table 3 lists the issues with persistent differences among various groups. The analysis of gender influence reveals a significant difference (*p* < 0.05) in perceptions of a female’s chest impact on CPR between males and females. A higher percentage of females express disagreement that a woman’s chest affects CPR performance, compared to males (Females: 21.59%, Males: 14.08%). Additionally, a notable proportion of proficient male CPR technicians remain neutral on this issue (20.89%).

**Table 3:** Analysis of the influence of gender, age and occupation on the choices of the population who are familiar with CPR

Question	Factor	Category	*n*	% of Group	W/*P* Value
Question 1*	Gender	Male	5	2.43%	W = 11,574, *P* = 0.027
		Female	15	6.61%	
	Age	18–35	9	3.04%	W = 15.318, *P* < 0.001
		36–50	5	5.95%	
		51–75	6	11.32%	
	Occupation	Non-medical	2	4.17%	W = 13.727, *P* = 0.001
		Medical worker	9	67.44%	
		Medical student	9	3.52%	
Question 2**	Gender	Male	24	11.65%	
		Female	49	21.59%	
	Age	18–35	47	15.88%	W = 34.474, *P* < 0.001
		36–50	19	22.62%	
		51–75	7	13.21%	
	Occupation	Non-medical	5	10.42%	W = 22.734, *P* < 0.001
		Medical worker	30	6.98%	
		Medical student	38	14.84%	
Question 3***	Gender	Male	43	20.87%	W = 6.0587, *P* = 0.049
		Female	29	12.78%	
	Age	18–35	54	18.24%	
		36–50	9	10.71%	
		51–75	9	16.98%	
	Occupation	Non-medical	4	8.33%	W = 8.0128, *P* = 0.018
		Medical worker	20	20.93%	
		Medical student	19	7.42%	

* Do you believe that the female breast would impede your performance of CPR?

** Do you believe that performing CPR on males is more challenging than on females?

*** Would you consider the patient’s privacy and cover their exposed chest while administering CPR?

The analysis of age influence on the CPR-trained population reveals a significant difference in agreement levels among age groups for questions about the impact of a woman’s chest on CPR, the difficulty of CPR on males versus females, and patient privacy during CPR. The 18–35 age group has notably lower agreement compared to the 36–50 and 51–75 age groups. The analysis of identity and occupation influence shows significant differences in agreement among different groups for the same questions. Medical students have significantly lower agreement on these questions than non-medical professionals and healthcare practitioners.

#### The importance of female models in CPR skills training

The proportion of individuals exposed to female mannequin models is very low across all demographics, with no significant difference between genders. Exposure is highest among the 18–35 age group (16.22%) and increases with higher education levels, reaching 38.10% among postgraduate degree holders. Medical students (16.8%) have the highest interaction with female manikins, followed by medical industry workers (11.63%) and non-medical workers (2.08%). The expectation for including female mannequin models in CPR training is very high, particularly among females (87.22%). Detailed proportions are available in Tables 4 and 5.

**Table 4:** Analysis on whether different groups have interacted with female mannequin models

Statistical variables	Yes		No		Total
	*n*	% of group	*n*	% of group	
Gender					
Male	24	11.65%	182	88.35%	206
Female	35	15.42%	192	84.58%	227
Age					
18–35 years 36–50 years 51–75 years	48	16.22%	248	83.78%	296
	10	11.90%	74	88.10%	84
	1	1.89%	52	98.11%	53
Occupation					
Medical students Medical industry workers Non-medical industry workers	43	16.80%	213	83.20%	256
	15	11.63%	114	88.37%	129
	1	2.08%	47	97.92%	48
Education Level					
Middle School Bachelor’s Degree Postgraduate and above	0	0.00%	13	100.00%	13
	51	13.25%	334	86.75%	385
	8	38.10%	13	61.90%	21

**Table 5:** Statistical assessment of various groups’ desire to augment the use of female mannequin models

Statistical variables	1		2		3		4		5		Total
	*n*	% of group	*n*	% of group	*n*	% of group	*n*	% of group	*n*	% of group	
Gender											
Male	7	3.40%	11	5.34%	33	16.02%	96	46.60%	59	28.64%	206
Female	5	2.20%	7	3.08%	17	7.49%	114	50.22%	84	37.00%	227
Age											
18–35 years 36–50 years 51–75 years	0	0.00%	4	1.35%	36	12.16%	152	51.35%	104	35.14%	296
	5	5.95%	7	8.33%	8	9.52%	38	45.24%	26	30.95%	84
	7	13.21%	7	13.21%	6	11.32%	20	37.74%	13	24.53%	53
Occupation											
Medical students Medical industry workers Non-medical industry workers	0	0.00%	4	1.56%	34	13.28%	127	49.61%	91	35.55%	256
	10	7.75%	9	6.98%	14	10.85%	56	43.41%	40	31.01%	129
	2	4.17%	5	10.42%	2	4.17%	27	56.25%	12	25.00%	48
Education Level											
Middle School Bachelor’s Degree Postgraduate and above	0	0.00%	1	7.69%	1	7.69%	8	61.54%	3	23.08%	13
	11	2.86%	15	3.90%	48	12.47%	185	48.05%	126	32.73%	385
	1	2.86%	2	5.71%	1	2.86%	17	48.57%	14	40.00%	35

## Discussion

Despite advancements in modern medical technology, progress in early prevention of sudden cardiac death is limited. However, due to its sudden onset and short warning time, the mortality rate remains unacceptably high [19]. OHCA ranks as the third leading cause of death in industrialized countries [20]. Bystander CPR is crucial for OHCA patient survival. The rates of survival to hospital discharge, one-month survival, and one-year survival are significantly higher in patients who receive bystander CPR compared to those who do not. Females with sudden cardiac arrest receive less effective bystander CPR than males, as studies suggest [21, 22]. This article investigates factors contributing to this disparity via a questionnaire survey.

According to the questionnaire results, factors such as gender, age, occupation, and education influence the public’s willingness to perform CPR on women in emergencies. To understand these influences, we analyzed significant differences among populations based on these factors for each question. We compiled questions with significant differences into a table and analyzed response variations across different populations, as well as potential reasons for these variations.

In emergency situations, women show a higher willingness to perform CPR on other women than men are on women. Further analysis of survey responses from different genders reveals disparities in attitudes and awareness towards administering CPR to female patients. Previous studies have indicated that gender is one of the significant factors influencing decision-making in emergency care in China [23]. Our findings are consistent with this trend, as only 29.61% of male participants considered it neither awkward nor offensive to remove a woman’s clothing during the CPR process. In traditional Chinese society, the teachings that emphasize maintaining gender boundaries lead most Chinese people to believe that kissing a stranger of the opposite gender in public is impolite, which may result in hesitation in providing cross-gender cardiopulmonary resuscitation [24]. Furthermore, within China’s societal norms, women are often regarded as a vulnerable group. Consequently, China has enacted the Law on the Protection of Women’s Rights and Interests, which includes specific provisions addressing sexual harassment [25]. In Chinese society, Confucianism, a fundamental aspect of traditional culture, exerts a profound influence. Traditional beliefs such as “distinction between men and women” and " it is improper for men and women to touch each other’s hand in passing objects” render Chinese individuals particularly sensitive to intimate interactions with the opposite sex [26–29]. As a result, men may be exceedingly cautious about physical contact with women during emergency medical situations. These factors may contribute to women receiving less effective CPR assistance from men, potentially explaining the disparity in assistance.

In emergencies, individuals aged 18–35 and 36–50 are more likely to perform CPR on women than those aged 51–75. Older individuals tend to prioritize privacy and respect, leading to a cautious approach in performing CPR, especially concerning actions such as undressing and chest contact [30].Younger individuals are generally more open-minded and accepting of bodily exposure and gender-related issue [31], but they may lack urgency and experience in rescue operations compared to older individuals. The 51–75 age group shows a slightly higher approval rate for immediately performing CPR, indicating better first aid awareness than the 36–50 age group. However, older individuals may refrain from assisting females due to conservative beliefs. Postgraduate and undergraduate degree holders are more willing to perform CPR on women than those with a high school education. This finding aligns with previous research on the Jordanian population, which shows that people with a higher educational level may show less concern about gender, be more willing to undress for cardiopulmonary resuscitation, and have a deeper understanding of the correct implementation of cardiopulmonary resuscitation [32].

In emergencies, healthcare professionals and those in non-healthcare industries are more willing to perform CPR on females compared to medical students. Despite having received the latest cardiopulmonary resuscitation training, medical students often hesitate due to lack of confidence in administering it, and they are reluctant to carry out first aid for the concern that their operations may not be standardized enough [33]. They may also be overly cautious about causing harm, leading to hesitation in emergencies. Additionally, their limited practical experience and relative youth compared to seasoned professionals may contribute to their hesitation. Both medical and non-medical professionals may hesitate due to conservative attitudes and rusty CPR skills, while medical students may also hesitate due to insufficient practical experience and excessive concern about risks. This hesitation among all groups may contribute to women having fewer opportunities to receive effective CPR.

In CPR education and training, many individuals have never interacted with female mannequin models. Although medical students undergo current CPR training, only 16.80% have encountered female mannequin models. However, there’s a widespread expectation for future CPR training to include female mannequin models, especially among females. The absence of such models may contribute to limited opportunities for females to receive effective CPR [13].

Within the cohort of individuals with professional CPR training and proficiency, many distinctions among individuals fade, suggesting that biases can be mitigated through CPR knowledge dissemination. This phenomenon may stem from the enhanced identification with the professional role of a “rescuer” after receiving training, which boosts confidence and decisiveness in emergencies. This sense of identity and confidence may outweigh other factors like gender or occupation, reducing the impact of these variables [34].

In conclusion, the limited opportunities for women to receive effective CPR may stem from factors such as gender discrimination, societal traditional beliefs, and inadequate CPR training (including the absence of female mannequin models and insufficient publicity). To address this issue, future efforts should focus on enhancing public education to eliminate gender biases, improving the CPR training system by incorporating more female mannequin models to familiarize trainees with physiological differences in women, and ensuring they master essential CPR techniques for females. Moreover, expanding CPR knowledge dissemination, raising public awareness of emergency assistance, and enabling more individuals to acquire CPR skills are crucial. Additionally, refining emergency medical assistance laws and regulations to protect rescuers’ rights and alleviate public concerns is essential [35, 36].

This research has several limitations. Firstly, due to the constraints of the research design at that time, we lacked multivariate statistical methods to control confounding variables, and complex factors such as educational level, age, and prior CPR training might have influenced the observed differences. Secondly, the cohort comparison between individuals who underwent CPR training and those who did not does not account for potential modifiers of training efficacy (e.g., frequency of practice, professional experience), which requires further investigation. Additionally, this sample was mainly collected from medical colleges in southeastern China, which may have introduced selection bias, with an excessive representation of medically trained participants, potentially underestimating the willingness of bystanders in the non-medical population. Moreover, the data collected in this study is limited to China, which restricts the generalizability to other cultures and regions. Future research should be geographically and culturally expanded to include larger and more diverse samples (including non-medical bystanders) and employ advanced multivariate techniques to validate these findings and explore regional or sociocultural influences on CPR-related gender differences.

## Conclusion

This study has shed light on the disparity in bystander CPR provision between male and female patients experiencing sudden cardiac arrest by web-based survey. The survey results have revealed that a complex interplay of factors, including gender discrimination, societal traditional beliefs, and inadequate CPR training, contributes to the decreased likelihood of women receiving effective CPR in emergency situations. These findings may offer guidance for the formulation of targeted intervention strategies to enhance the rate at which women receive effective CPR.

## Data Availability

Data is provided within the manuscript or supplementary information files.
